# Mechanotransduction: Tuning Stem Cells Fate

**DOI:** 10.3390/jfb2020067

**Published:** 2011-06-21

**Authors:** Francesco D'Angelo, Roberto Tiribuzi, Ilaria Armentano, Josè Maria Kenny, Sabata Martino, Aldo Orlacchio

**Affiliations:** 1 Department of Experimental Medicine and Biochemical Science, Section of Biochemistry and Molecular Biology, University of Perugia, Via del Giochetto, 06126 Perugia, Italy;E-Mails: francescodangelo@hotmail.it (F.A.); robertotiribuzi@yahoo.it (R.T.); 2 Materials Engineering Centre, UdR INSTM, NIPLAB, University of Perugia, Strada di Pentima 4, 05100 Terni, Italy; E-Mails: Ilaria.armentano@unipg.it (I.A.); jkenny@unipg.it (J.M.K.); 3 Institute of Polymer Science and Technology, CSIC, Juan de la Cierva 3, 28006 Madrid, Spain

**Keywords:** ESCs, ASCs, iPS, mechanotransduction, regenerative medicine, tissue engineering

## Abstract

It is a general concern that the success of regenerative medicine-based applications is based on the ability to recapitulate the molecular events that allow stem cells to repair the damaged tissue/organ. To this end biomaterials are designed to display properties that, in a precise and physiological-like fashion, could drive stem cell fate both *in vitro* and *in vivo*. The rationale is that stem cells are highly sensitive to forces and that they may convert mechanical stimuli into a chemical response. In this review, we describe novelties on stem cells and biomaterials interactions with more focus on the implication of the mechanical stimulation named mechanotransduction.

## 1. Regenerative Medicine

Therapy for incurable degenerative diseases is based on the replacement of damaged cells within the organs or tissues together with the restoration of the missing biological function. Thus, (i) novel technologies allow the development of high-tech devices that mimick the damaged organs [1,2]; (ii) gene therapy strategies allow the substitution of the defective gene with the corresponding healthy copy and re-establish the lost protein function [3,4]; (iii) finally stem cell transplantation allows the replacement of damaged cells and repairs the tissue/organ homeostasis [5,6,7].

Currently, tissue and organ replacement could be obtained by tissue engineering strategies. Stem cells and novel smart biomaterials are combined in some way so that they can regenerate or replace the tissue in the body. So far the regeneration of tissues such as cornea, skin and trachea represents some of the best-known examples of this approach [8,9,10,11,12]. Thus, Rama *et al.* [8,9] generated cohesive sheets of authentic corneal epithelium from autologous cultured limbal cells and restored the corneal surface of two patients with complete loss of the corneal-limbus epithelium [8,9]. Similarly, Macchiarini and co-workers produced a functional engineered trachea and indicated a successful way for the treatment of patients with serious clinical airway disorders [11]. Exiting results were obtained by Eiraku *et al.* [13], who showed the autonomous formation of the optic cup (retinal primordium) structure from a three-dimensional culture of mouse embryonic stem cell aggregates. For the first time, they reported extraordinary videos recording the formation of an embryonic mouse eye as a consequence of self-organizing three-dimensional cultures of embryonic stem cells [13,14].

Chen *et al.* [15] reported a successful attempt at transplanting stem cells in two patients with mucopolysaccharidosis, [15,16]. These authors also reported the improvement of heart conditions in these patients [17]. Other authors reported that bone marrow-derived c-kit^+^ stem cells therapy, improved cardiac function by the stimulation of endogenous cardiomyocytes progenitors [18].

## 2. Stem cells

Stem cells (SCs) are cells with the properties of self-renewal, indefinite proliferative potential and multipotential ability to give rise to different cell lineages.

Stem cell population homeostasis is widely thought to be achieved through peculiar cell division. Asymmetric cell division could results in a daughter that remains stem cell and a progenitor daughter, alternatively, symmetric cell division results in two stem cell daughters [19].

Within the niche, stem and progenitor cells use asymmetric cell divisions to balance proliferation and differentiation. This process is regulated by proteins asymmetrically distributed during mitosis, of which some confer polarity while others govern spindle positioning. In the developing mouse skin, progenitor cells execute a switch from symmetric to predominantly asymmetric divisions concomitant with stratification. Williams *et al.* [20] demonstrated that compromising asymmetric cell divisions lead to profound defects in stratification, differentiation and barrier formation [20]. Mouse intestinal stem cells divide symmetrically and adopt stem or differentiating states in a stochastic manner [21,22]. Their turnover follows a pattern of neutral drift dynamics, in which stochastic stem cell loss through differentiation is compensated by symmetric self-renewal of neighboring stem cells [21,22].

In the mammalian brain, stem cell niches are retained within the subventricular zone (SVZ) and the specific cytoarchitectural organization within the narrow adult neural stem cell niche is critical for maintaining stem cell populations, guiding cell fate decisions and, ultimately, regulating the regenerative potential of the niche [23,24]. In addition to the restraints imposed by niche cytoarchitecture, the neural stem cell niche is under the influence of a complex array of diffusible molecules, including growth factors and neurotransmitters [25,26,27,28,29,30,31,32,33]. Many factors appear to influence age-related decreases in neurogenesis, including a reduction in specific growth factors and telomerase levels, changes in cell-cycle modulators, and high levels of corticosteroids and inflammation [34,35,36,37,38,39,40].

### 2.1. Embryonic Stem Cells

The first expandable human embryonic stem cell (hESCs) culture, was successfully derived from the inner cell mass of blastocysts in 1998 [41] and represents a potentially unlimited source of cells for regenerative medicine and tissue engineering strategies. These cells maintain their undifferentiated state for at least 80 passages *in vitro* when grown using current published protocols [41,42]. They can be differentiated into cells from all three embryonic germ layers: (i) ectoderm: skin and neurons [43,44,45,46]; (ii) mesoderm: blood, cardiac cells, cartilage, endothelial cells, and muscle [47,48,49]; (iii) endoderm: pancreatic cells [50,51,52]. Interestingly, while ESCs, can form teratomas *in vivo, in vitro* generate embryoid bodies, which are cell aggregations that contain all three embryonic germ layers [53,54]. Cyclin-dependent kinase 1 (Cdk1) is indispensable for the early development of the embryos. Cdk1 expression is tightly correlated with the undifferentiated state of ES cells by maintaining the unique undifferentiated and self-renewing state of mouse ES cells [55], whereas Cdk1 has a crucial role in orchestrating a fine balance between cellular proliferation, cell death and DNA repair in hESCs [56]. Recently, it was shown that the transition of ES cell differentiation from the epiblast state into neuroectodermal progenitors specifically depends on the expression and activator functionality of Zfp521 [57].

### 2.2. Adult Stem Cells

Adult stem cells (ASCs) are multipotent stem cells that, under controlled conditions, may differentiate into various cells *in vitro* and *in vivo* [58,59,60,61,62]. ASCs have been isolated from bone marrow, cord blood, skeletal muscle, brain, cornea, tooth and skin among other tissues [63,64,65,66]. ASCs can self-renew and undergo multipotential differentiation, however they show a more restricted differentiation potential compared to ESCs [67,68,69,70]. The main function of ASCs, within the body, is their involvement in tissue repopulation under physiological and pathological conditions [71,72]. Cell-fate decisions in the developing embryo are orchestrated by a complex balance between cell-autonomous signals and stimuli from the surrounding micro-environment. Within the stem cells niche these processes control the birth and maturation of stem cells that replenish mature cells in adult tissues [73,74,75]. For instance, satellite cells are considered the main progenitors of adult skeletal muscle and present several stem cell properties [76]. Recently, d'Aquino *et al.* [77] isolated human neural crest derived postnatal cells, from the dental follicle, that exhibit remarkable embryonic features both *in vitro* and *in vivo*.

Notably, ASCs respond to microenvironment changes which, in turn, may alter their fate [73,74,75], suggesting ASCs useful for regenerative medicine applications. In this regard, mesenchymal stem cells (MSCs) remain the most promising type of adult stem cells for regenerative medicine in cell therapy and tissue engineering. Their most common sources are bone marrow, fat, amniotic fluid, amniotic membrane, and umbilical cord matrix [78,79,80].

### 2.3. Induced Pluripotent Stem Cells

Potential clinical applications of ES cells raise many practical and ethical concerns. In this regard, a major breakthrough was achieved in 2006, when it was shown that pluripotent stem cells could be obtained by transducing either mouse embryonic or adult fibroblasts with a limited set of specific transcription factors [81]. These reprogrammed cells, named induced pluripotent stem (iPS) cells, resembled ES cells in many of their characteristics. To date, iPS cells have been generated from cells of several species using different sets of reprogramming factors. For instance, reprogramming blood cells to iPSCs provides a novel tool for modeling blood diseases *in vitro*. Hu and co-workers [82] demonstrated that iPSCs free of transgene and vector sequences could be efficiently generated using non-integrating episomal vectors from human bone marrow and cord blood mononuclear cells of healthy donors and the bone marrow of a patient with chronic myeloid leukemia. This approach provides an opportunity to explore normal and diseased cord blood and bone marrow samples without any limitations associated with virus-based methods [82]. Moreover, somatic cells reprogramming to iPSCs can also be achieved using poly(β-amino ester)s as the transfection reagent for the delivery of a single CAG-driven polycistronic plasmid expressing Oct4, Sox2, Klf4, c-Myc and a GFP reporter gene (OSKMG) [83].

However, many questions regarding the molecular process of induced reprogramming remain unanswered and need to be addressed before iPS cells can be employed in the clinics. In fact, iPSCs cell-line-specific differences and the mechanisms regulating pluripotency must be better understood. Despite the common ability of hiPSCs and hESCs to differentiate into all 3 germ layers, their functional equivalence at the single cell level remains to be demonstrated. Comparison between single hESCs and single hiPSCs have indeed revealed a much higher heterogeneity in gene expression levels in affecting hiPSCs, suggesting that these cells feature an alternate, less stable pluripotent state [84,85].

Currently, patient-specific iPSCs are extensively studied for translational research applications. Yazawa and co-workers generated iPSCs from fibroblasts from Timothy syndrome patients, and differentiated these cells into cardiomyocytes. This study provides new opportunities for studying the molecular and cellular mechanisms of cardiac arhythmias in humans, and provides a robust assay for developing new drugs to treat these diseases [86].

Recently, it was reported the generation of iPS cells from peripheral blood CD34^+^ cells of two patients with myeloproliferative disorders (MPDs). The MPD-derived iPS cells, despite unchanged phenotypes, karyotype, and pluripotency, showed increased erythropoiesis and recapitulated features of primary CD34^+^ cells of the corresponding patient from whom the iPS cells were derived. These iPS cells provide a renewable cell source and a prospective hematopoiesis model for investigating MPD pathogenesis [87].

A comprehensive report published by Park *et al.* in 2008 [88] showed for the first time the feasibility of generating iPS cells from fibroblasts of patients with complex genetic disorders including Huntington and Parkinson disease, diabetes mellitus and Down syndrome [88]. Another example is spinal muscular atrophy (SMA), a neuromuscular disorder caused by mutations in the SMN1 gene that result in the degeneration of selected motorneurons. iPS cells established from a patient suffering SMA (iPS-SMA) maintain the disease phenotype and are capable of differentiating into motorneurons initially, however, these cells degenerate with time, unlike their counterpart derived from the patient's healthy mother [89,90].

## 3. Stem Cells and Biomaterial Interactions

The design of biomaterials with specific properties represents a valid approach to modulate and control the stem cell environment. Nanotechnology enables the development of new systems that mimic the complex hierarchical structure of the native tissue. Therefore, nanotechnology and biology based rationales would be capable to address several biomedical problems, and revolutionize medicine. The physical properties as well as the chemical properties of materials, including size, shape, mechanical properties, surface texture, *etc.* can regulate biological responses and provide mechanical stimuli to stem cells [91].

**Figure 1:**
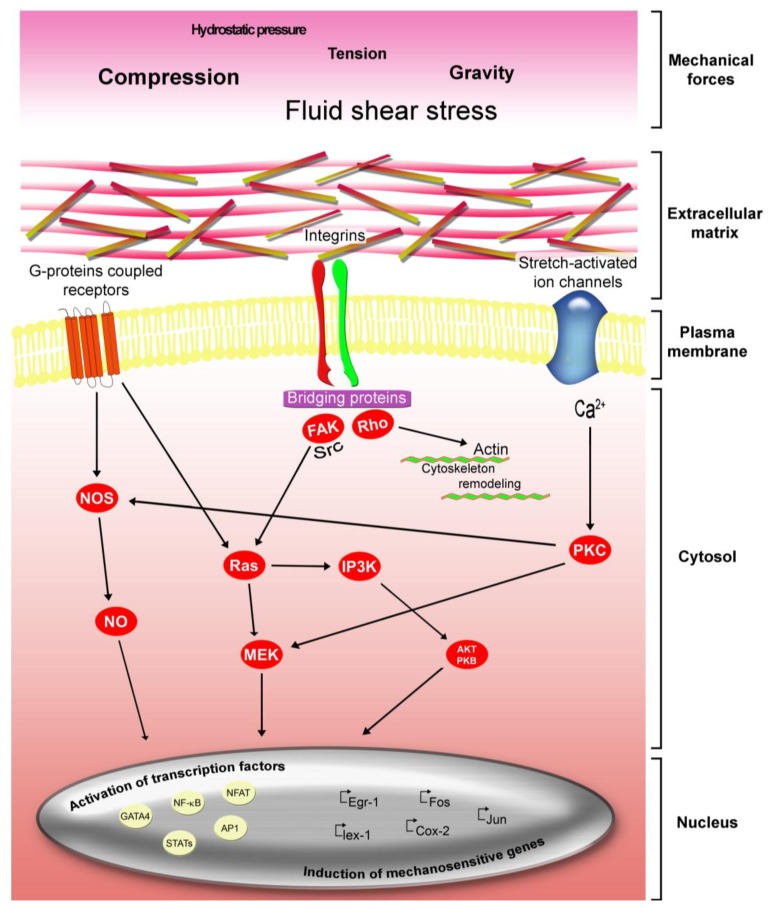
Stem cells respond to different mechanical forces loading by activating multiple intracellular signaling pathways that are implicated in the maintenance and regulation of cellular functions. Stem cells can sense the mechanical loading through a diverse group of membrane-anchored mechanosensors (stretch-activated ion channels, cell-membrane-spanning G-protein-coupled receptors, and integrins). This mechanical stimulus is then converted to biochemical signals by triggering the multi-step activation of downstream partners in an array of signaling cascades in the cytoplasm. The convergence of these pathways results in the activation of select transcription factors, including nuclear factor-B (NF-κB) and nuclear factor of activated T cells (NFAT), which then translocate to the nucleus and modulate the expression of a panel of mechanosensitive genes, including *Egr1* and *lex1*.

Mechanical forces (e.g., gravity, tension, compression, hydrostatic pressure, and fluid shear stress) influence the growth and shape of every tissue and organ under physiological and pathological conditions [92]. Additionally, traction forces generated by cells may markedly influence many biological processes such as self-renewal and differentiation. Research is focused on the identification of critical mechanosensitive molecules and cellular components that contribute to the mechanotransduction response [93,94]. The presence of isometric tension (prestress) at all levels of these multiscale networks ensures that various molecular scale mechanochemical transduction mechanisms proceed simultaneously and produce a concerted response. Future research in this area will therefore require a better understanding of tensionally integrated (tensegrity) systems of mechanochemical controls [95,96,97]. Highly coordinated extensive cellular components including cytoskeleton, adhesion complexes, and ion channels have been implicated as the primary mediators of mechanotransduction (Figure 1) [64,98,99,100,101,102,103,104,105,106,107,108] suggesting that, the generation of successful tissue engineering implants depend on the control of mechanical forces [109,110]. For instance, it has been demonstrated that nanoscale topographies were able to stimulate human MSCs to produce bone mineral *in vitro,* in the absence of osteogenic supplements, and with efficiency comparable to that of cells cultured with osteogenic media [111]. Moreover, a recent advance made in the tissue engineering field is the generation of selective differentiation of MSCs into specific cells phenotype by applying various mechanical forces using matrix stiffness or topography [101,112,113,114,115]. In this regard we have showed that human MSCs responded to hydrogenated amorphous carbon (a-C:H) nanotopographies with groove or grid surface structures, inducing specific changes of their microtubule organization. In particular, we observed that the groove nanopatterns exerted a more dynamic effect, associated with stem cell alignment and elongation [64]. Moreover we demonstrated that the surface topography with micropatterned nanogrooves width/spacing of 40/30μm induced hMSCs to acquire neuronal characteristics in the absence of differentiating agents. These results were further validated by the observation that alternative a-C:H groove dimensions tested (80/40μm and 30/20μm) failed to induce stem cell differentiation [101].

It is likely that both chemical and topographical properties of material surfaces can influence cellular behavior and can control cell shape, functions and motility [103,104,105]. In this context, we have reported that radiofrequency oxygen plasma treatment was effective in changing the surface properties of Polylactide (PLLA) scaffolds. The treatment functionalized the surface of the PLLA homogeneously without affecting its bulk properties, changing wettability, roughness and the interaction of proteins with the surface of PLLA polymer and improving the stem cell attachment [104]. In the last two decades, nanocomposites have emerged as an efficient strategy to changes mechanical, thermal electrical properties of polymers, in order to prepare new biomaterials with enhanced properties. Engineered synthetic polymeric nanocomposites can allow precise and systematic control over the mechanical properties of the cell substrate, and provided quantitative information about the forces that are sensed and exerted by cells [104,106]. The effect of matrix stiffness on the phenotype and differentiation pathway of MSC was reported by several groups showing that stem cells differentiated into neural, myogenic or osteogenic phenotypes depending on whether they were cultured on two-dimensional (2D) substrates of elastic moduli in the lower (0.1–1 kPa), intermediate (8–17 kPa) or higher ranges (34 kPa). Similar results were found for the three-dimensional (3D) culture [107]. Notable, the cellular response to matrix stiffness may be very different in different cell types and depends on the nature of the adhesion receptor by which the cell binds its substrate [108].

### 3.1. Integrins, Cytoskeleton Involvements in Mechanotransduction

Integrins, acting as mechanosensors alone or in concert with cytoskeletal proteins, are one of the major components involved in mechanotransduction [116,117,118,119,120]. Integrins, of adherent cells (*i.e.*, MSCs), are considered direct mechanosensors that physically connect the ECM to the cytoskeleton [121], thus acting as signaling receptors. More in particular, cell integrins bind the ECM externally, while linking the focal adhesion to actin cytoskeleton [122]. Moreover, mechanical forces promote the assembly of focal adhesion spots (FAs), by altering the relative positions of specific internally FAs components (such as vinculin and fibronectin) and their conformations, and trigger intergrin-dependent signaling and MAPKs activation [92,122,123,124].

Tension transmission to the nucleus is due to the actin cytoskeleton, and those tensional forces along the actomyosin contractile system are also regulated by the degree of phosphorylation of the myosin light chain [125,126]. In fact, as demonstrated by Chrzanowska-Wodnicka and Burridge in 1996 [127], the activation of RhoA controls the development of FAs and stress fibers, whereas the influence of RhoA on actin cytoskeleton is mediated by its downstream effector ROCK, which inactivate myosin phosphatase, thereby on inducing the stabilization of filamentous actin and the stress fiber formation [128]. The remodeling of the microfilament and microtubule networks occur as consequence of mechanical stretching, while the prevention of cytoskeletal remodeling mitigates stretch-induced increase in gene transfer and expression [129], thereby eliciting “local” cellular events. A model of cell structure suggests that this dynamic remodeling of the cytoskeleton is also a ‘hard-wired’ tensegrity [130]. This model takes into account the observation that individual cytoskeletal filaments can bear significant tensile and compressive loads in living cells because their structural integrity is maintained for longer than the turnover time of individual protein monomers [131,132,133]. In particular mechanical forces applied to the cell surface mainly involve integrins and cadherins that are physically coupled to cytoskeletal filament networks and, in turn, are linked to nuclear scaffolds, nucleoli, chromatin and DNA inside the nucleus [134,135]. This raises the intriguing possibility that mechanical forces applied the cell surface might act by promoting mechanochemical conversion in the nucleus [120], in addition to mechanochemical transduction in the cytoplasm [136].

Although the molecular mechanism by which a mechanical stimulus is translated into a chemical response in biological systems is still unclear, the mechanical stretching of single cytoplasmic proteins is known to activate binding of other molecules. For example, the application of physiologically relevant forces cause stretching of single talin rods that expose cryptic binding sites for vinculin. Thus, in the talin-vinculin system, molecular mechanotransduction can occur by protein binding after exposure of buried binding sites in the talin-vinculin system. Such protein stretching may be a more general mechanism for force transduction [94].

Compressive stimulation increases the level of phosphorylated focal adhesion kinase (FAK) and prostaglandin E(2) production. The FAK-integrin complex plays a role in mechanoreception and mechanotransduction in human periodontal ligament cells [137]. In these, it was demonstrated that strain-dependent mechano-/signal-transduction also involves MAP-kinases p42/44, and p38 stress kinase in conjunction with the amount of MMP-13, and integrin subunits beta1 and beta3 [138].

Mammographically dense breast tissue is one of the greatest risk factors for developing breast carcinoma, but the molecular mechanisms still remain largely unknown. Recently, it was proposed that chronically elevated signaling loop (FA-FAK-Rho) is necessary to generate and maintain the invasive phenotype. Moreover, this signaling network resulted in hyperactivation of the Ras-mitogen-activated protein kinase (MAPK) pathway, which activated a clinically relevant proliferation signature that predicts patient outcome. In this scenario, these findings provide compelling evidence of the importance of mechanical features of the microenvironment, and suggest that mechanotransduction in these cells occurs through a FAK-Rho-ERK signaling network with extra cellular signal-regulated kinase (ERK) as a bottleneck through which much of the response to mechanical stimuli is regulated [139].

Consequences of mechanical forces applied to cell surface are changes in Ca^2+^ influx through stretch-activated channels [140]. This alteration in the Ca^2+^ influx could result in intracellular activation of several molecules, such as NF-κB, cAMP-response element binding protein (CREB), membrane kinases and EGFR, leading to the activation of MAPK signaling pathway [141,142,143].

Both mechano-responsive elements (adhesion-dependent and ion channel-based mechanism) are linked via a motor protein (such as myosin II) to the cytoskeleton inside the cell and to an extra cellular anchor (usually the ECM).

Mechanotransduction could also act at the cell membrane level, through the involvement of G-protein-coupled receptors (GPCRs) [144,145,146].

### 3.2. Mechanical Forces with Asymmetrical Direction

Mechanical forces with a clear direction (such as the circumferential stretch of the arterial tree) cause only transient molecular signaling of pro-inflammatory and proliferative pathways, which become down-regulated when such directed mechanical forces are sustained. In contrast, mechanical forces without a definitive direction (e.g., disturbed flow and relatively undirected stretch) cause sustained molecular signaling of pro-inflammatory and proliferative pathways. The vascular endothelial cells (EC) responses to directed mechanical stimuli involve the remodeling of EC structure to minimize alterations in intracellular stress/strain and elicit adaptive changes in EC signaling as a result of sustained stimuli; these cellular events constitute a feedback control mechanism to maintain vascular homeostasis and are atheroprotective. Such a feedback mechanism does not operate effectively in regions of complex geometry, where the mechanical stimuli do not have clear directions, thus placing these areas at risk for atherogenesis [92,147].

Mechanical forces associated with blood flow are determinants of vascular morphogenesis and physiology. Recent data highlighted the endothelial mechanotransducers that might mediate responses to blood flow, the effects of atheroprotective rather than atherogenic flow, the mechanisms that contribute to the progression of the disease and how systemic factors interact with flow patterns to cause atherosclerosis [148].

The immunoglobulin family receptor platelet endothelial cell adhesion molecule (PECAM)-1, vascular endothelial cell cadherin (VE-cadherin) and vascular endothelial growth factor receptor 2 (VEGFR2) comprise a mechanosensory complex capable of conferring responsiveness to flow in heterologous cells. In support of the relevance of this pathway *in vivo*, PECAM-1-knockout mice do not activate NF-κB and downstream inflammatory genes in regions of disturbed flow. Therefore, this mechanosensing pathway is required for the earliest events associated with the development of atherogenesis [149].

Another example is reported in the study by Shi and collaborators [150] that proposed a conceptual mechanotransduction model for interstitial flow, wherein cell surface glycocalyx HSPGs, in the presence of integrin-mediated cell-matrix adhesions and cytoskeleton organization, sense interstitial flow and activate the FAK-ERK signaling axis, leading to upregulation of MMP expression and cell motility in 3D [150].

The mechanotransduction-induced EC adaptive processes in the straight part of the aorta represent a case of the “Wisdom of the Cell”, as a part of the more general concept of the “Wisdom of the Body” raised by Cannon, regarding the maintenance of cellular homeostasis in the presence of external perturbations [147].

Additionally, a new role for caveolae as a physiological membrane reservoir that quickly accommodates sudden and acute mechanical stresses has been recently proposed by Sinha and co-workers [151]. Acute mechanical stress induced by osmotic swelling or by uniaxial stretching results in a rapid disappearance of caveolae, a reduced caveolin/Cavin1 interaction, and an increase of free caveolins on the plasma membrane. The absences of a functional caveola reservoir in myotubes from muscular dystrophic patients enhance membrane fragility under mechanical stress [151]. Mechanical forces are also critical for fetal lung development, as showed by Wang and co-workers, who demonstrated that Caveolin-1 is present in E19 fetal type II epithelial cells, and is translocated from the plasma membrane to the cytoplasm by mechanical stretch and functions as an inhibitory protein in stretch-induced type II cell differentiation via the ERK pathway [152].

### 3.3. Wnt and Beta-Catenin Involvement in Mechanotransduction

The normal translocation of beta-catenin to the nucleus in osteoblasts that is induced by oscillatory fluid shear stress (OFSS) is enhanced when the nucleocytoplasmic shuttling Nmp4/CIZ (transcription factor nuclear matrix protein-4/cas interacting zinc finger protein) is absent. Furthermore, other aspects of OFSS-induced mechanotransduction, generally associated with the beta-catenin signaling pathway, including ERK, Akt, and GSK3beta activity, as well as expression of the beta-catenin-responsive protein cyclin D1, are also enhanced in cells lacking Nmp4/CIZ. Finally, in the absence of Nmp4/CIZ, OFSS-induced cytoskeletal reorganization and the formation of focal adhesions between osteoblasts and the extra cellular substrate is qualitatively enhanced, suggesting that Nmp4/CIZ may reduce the sensitivity of bone cells to mechanical stimuli. Together these results support the notion that Nmp4/CIZ plays an inhibitory role in the response of bone cells to mechanical stimulation induced by OFSS [153].

Recent findings indicate a stimulating role of Wnt signaling in bone mechanotransduction. In fact, Jansen and co-worker [154] demonstrated a biphasic effect of mechanical loading on beta-catenin in mineralizing human differentiating osteoblasts independent on the ERK pathway. Moreover, the authors hypothesized that the biphasic aspect of Wnt/beta-catenin signaling with a strong decrease up to 40 h after the stretch induction, is important for the anabolic effects of mechanical stretch on bone [154].

Other finding highlighted the involvement of nitric oxide, focal adhesion kinase, and the phosphatidyl inositol-3 kinase/Akt signaling pathway in beta-catenin pathway activation. Authors found that mechanical stimulation by pulsating fluid flow (PFF) induced beta-catenin stabilization and activation of the Wnt/beta-catenin signaling pathway. This stabilization of beta-catenin and activation of the beta-catenin signaling pathway PFF-induced was abolished by adding focal kinase inhibitor FAK inhibitor-14, or phosphatidyl inositol-3 kinase inhibitor LY-294002. Addition of nitric oxide synthase inhibitor L-NAME also abolished PFF-induced stabilization of beta-catenin This suggests that mechanical loading activates the beta-catenin signaling pathway by a mechanism involving nitric oxide, focal adhesion kinase, and the Akt signaling pathway [155].

Liedert and co-workers [156] investigated regulatory mechanisms by which mechanical loading exerts its role in bone mass homeostasis [156]. They demonstrated that estradiol (E2) had a sensitizing effect on mechanically induced cyclooxygenase-2 (Cox-2) expression, which seemed to be ligand-specific in that was abolished by using the anti-estrogen ICI182, 780. However, mechanical strain in the presence of Wnt signaling activators decreased both the E2 sensitizing effect and the stimulatory effect of Wnt signaling in the absence of strain [154,156].

## 4. Future Perspectives

Despite an overall scepticism, scientists and clinicians believe that tissue engineering approaches could be a powerful therapeutic opportunity in clinical practice. Although, the state-of-the-art for stem-cell-biomaterial clinical trials is still at an early stage and the relevant functional outcomes have yet proven successful promising tissue engineering scaffolds have been developed for skin, cornea, bone and trachea [8,9,10,11,12,13,157]. Moreover, the discovery of iPS cells as patient-specific stem cells represents a breakthrough for the stem cell-based therapy and basic cell biology [81,157]. This is reflected by the accessibility to patient specific iPS cells, which also allows researchers to investigate the pathogenesis of the disease *in vitro* [88,158,159,160]. Tissue engineering presents an enormous opportunity for developmental biology and basic research, as well as drug delivery and personalized therapies.

Furthermore, nanotechnologies may allow the understanding of molecular mechanisms of mechano-sensing and -transduction, and thus solve key questions in tissue engineering strategies. To this end, collaborative efforts between clinicians, biologists and materials scientists become critical for answering key biological questions and promoting interdisciplinary stem-cell research towards clinical relevance.

In conclusion, the future of regenerative medicine is based on the fabrication of innovative devices that take into account the feedback between stem cells biology, cell sensing of force, and biomaterials' properties (topography, stiffness, electrical conductibility, drugs release and form).

## References

[1] Orlacchio A., Bernardi G., Orlacchio A., Martino S. (2010). Stem cells and neurological diseases. Discov. Med..

[2] Lindvall O., Kokaia Z. (2010). Stem cells in human neurodegenerative disorders-time for clinical translation?. J. Clin. Invest..

[3] Martino S., Marconi P., Tancini B., Dolcetta D., De Angelis M.G., Montanucci P., Bregola G., Sandhoff K., Bordignon C., Orlacchio A. (2005). A direct gene transfer strategy via brain internal capsule reverses the biochemical defect in Tay-Sachs disease. Hum. Mol. Genet..

[4] Martino S., di Girolamo I., Orlacchio A., Datti A., Orlacchio A. (2009). MicroRNA implications across neurodevelopment and neuropathology. J. Biomed. Biotechnol..

[5] Martino S., di Girolamo I., Cavazzin C., Tiribuzi R., Galli R., Rivaroli A., Valsecchi M., Sandhoff K., Sonnino S., Vescovi A. (2009). Neural precursor cell cultures from GM2 gangliosidosis animal models recapitulate the biochemical and molecular hallmarks of the brain pathology. J. Neurochem..

[6] Gentner B., Visigalli I., Hiramatsu H., Lechman E., Ungari S., Giustacchini A., Schira G., Amendola M., Quattrini A., Martino S. (2010). Identification of hematopoietic stem cell-specific miRNAs enables gene therapy of globoid cell leukodystrophy. Sci. Transl. Med..

[7] Lattanzi A., Neri M., Maderna C., di Girolamo I., Martino S., Orlacchio A., Amendola M., Naldini L., Gritti A. (2010). Widespread enzymatic correction of CNS tissues by a single intracerebralinjection of therapeutic lentiviral vector in leukodystrophy mouse models. Hum. Mol. Genet..

[8] Rama P., Matuska S., Paganoni G., Spinelli A., de Luca M., Pellegrini G. (2010). Limbal stem-cell therapy and long-term corneal regeneration. N Engl. J. Med..

[9] Rama P., Bonini S., Lambiase A., Golisano O., Paterna P., de Luca M., Pellegrini G. (2001). Autologous fibrin-cultured limbal stem cells permanently restore the corneal surface of patients with total limbal stem cell deficiency. Transplantation.

[10] Pellegrini G., Ranno R., Stracuzzi G., Bondanza S., Guerra L., Zambruno G., Micali G., de Luca M. (1999). The control of epidermal stem cells (holoclones) in the treatment of massive full-thickness burns with autologous keratinocytes cultured on fibrin. Transplantation.

[11] Macchiarini P., Jungebluth P., Go T., Asnaghi M.A., Rees L.E., Cogan T.A., Dodson A., Martorell J., Bellini S., Parnigotto P.P. (2008). Clinical transplantation of a tissue-engineered airway. Lancet.

[12] Pellegrini G., Traverso C.E., Franzi A.T., Zingirian M., Cancedda R., de Luca M. (1997). Long-term restoration of damaged corneal surfaces with autologous cultivated corneal epithelium. Lancet.

[13] Eiraku M., Takata N., Ishibashi H., Kawada M., Sakakura E., Okuda S., Sekiguchi K., Adachi T., Sasai Y. (2011). Self-organizing optic-cup morphogenesis in three-dimensional culture. Nature.

[14] Ali R.R., Sowden J.C. (2011). Regenerative medicine: DIY eye. Nature.

[15] Chen J., Jiang H., Dong L., Wang Y., Luo C., Zhou M., Zhang W., Huang S., Gu X., Qiu W. (2008). Treatment of 2 children with mucopolysaccharidosis by allogeneic hematopoietic stem cell transplantation. Zhonghua Yi Xue Yi Chuan Xue Za Zhi.

[16] Yeung A.H., Cowan M.J., Horn B., Rosbe K.W. (2009). Airway management in children with mucopolysaccharidoses. Arch. Otolaryngol. Head Neck. Surg..

[17] Roomans G.M. (2010). Tissue engineering and the use of stem/progenitor cells for airway epithelium repair. Eur. Cell. Mater..

[18] Loffredo F.S., Steinhauser M.L., Gannon J., Lee R.T. (2011). Bone marrow-derived cell therapy stimulates endogenous cardiomyocyte progenitors and promotes cardiac repair. Cell. Stem. Cell..

[19] Baumann K. (2010). Dividing with symmetry. Nat. Rev. Mol. Cell. Biol..

[20] Williams S.E., Beronja S., Pasolli H.A., Fuchs E. (2011). Asymmetric cell divisions promote Notch-dependent epidermal differentiation. Nature.

[21] Snippert H.J., van der Flier L.G., Sato T., van Es J.H., van den Born M., Kroon-Veenboer C., Barker N., Klein A.M., van Rheenen J., Simons B.D. (2010). Intestinal crypt homeostasis results from neutral competition between symmetrically dividing Lgr5 stem cells. Cell.

[22] Lopez-Garcia C., Klein A.M., Simons B.D., Winton D.J. (2010). Intestinal stem cell replacement follows a pattern of neutral drift. Science.

[23] Fuchs E., Tumbar T., Guasch G. (2004). Socializing with the neighbors: stem cells and their niche. Cell.

[24] Conover J.C., Notti R.Q. (2008). The neural stem cell niche. Cell Tissue Res..

[25] Anderson M.F., Aberg M.A., Nilsson M., Eriksson P.S. (2002). Insulin-like growth factor-I and neurogenesis in the adult mammalian brain. Brain Res. Dev. Brain Res..

[26] Conover J.C., Allen R.L. (2002). The subventricular zone: new molecular and cellular developments. Cell Mol. Life Sci..

[27] Doetsch F. (2003). A niche for adult neural stem cells. Curr. Opin. Genet Dev..

[28] Hagg T. (2005). Molecular regulation of adult CNS neurogenesis: An integrated view. Trends Neurosci..

[29] Petersen P.H., Zou K., Hwang J.K., Jan Y.N., Zhong W. (2002). Progenitor cell maintenance requires Numb and Numblike during mouse neurogenesis. Nature.

[30] Temple S. (2001). The development of neural stem cells. Nature.

[31] Lim D.A., Alvarez-Buylla A. (1999). Interaction between astrocytes and adult subventricular zone precursors stimulates neurogenesis. Proc Natl. Acad. Sci. USA..

[32] Lennington J.B., Yang Z., Conover J.C. (2003). Neural stem cells and the regulation of adult neurogenesis. Reprod. Biol. Endocrinol..

[33] Maric D., Fiorio, Pla A., Chang Y.H., Barker J.L. (2007). Self-renewing and differentiating properties of cortical neural stem cells are selectively regulated by basic fibroblast growth factor (FGF) signaling via specific FGF receptors. J. Neurosci..

[34] Brazel C.Y., Rao M.S. (2004). Aging and neuronal replacement. Ageing Res. Rev..

[35] Enwere E., Shingo T., Gregg C., Fujikawa H., Ohta S., Weiss S. (2004). Aging results in reduced epidermal growth factor receptor signaling, diminished olfactory neurogenesis, and deficits in fine olfactory discrimination. J. Neurosci..

[36] Tropepe V., Craig C.G., Morshead C.M., van der Kooy D. (1997). Transforming growth factor-alpha null and senescent mice show decreased neural progenitor cell proliferation in the forebrain subependyma. J. Neurosci..

[37] Caporaso G.L., Lim D.A., Alvarez-Buylla A., Chao M.V. (2003). Telomerase activity in the subventricular zone of adult mice. Mol. Cell Neurosci..

[38] Blackburn E.H. (2001). Switching and signaling at the telomere. Cell.

[39] Smogorzewska A., de Lange T. (2004). Regulation of telomerase by telomeric proteins. Annu. Rev. Biochem..

[40] Sarin K.Y., Cheung P., Gilison D., Lee E., Tennen R.I., Wang E., Artandi M.K., Oro A.E., Artandi S.E. (2005). Conditional telomerase induction causes proliferation of hair follicle stem cells. Nature.

[41] Thomson J.A., Itskovitz-Eldor J., Shapiro S.S., Waknitz M.A., Swiergiel J.J., Marshall V.S., Jones J.M. (1998). Embryonic stem cell lines derived from human blastocysts. Science.

[42] Reubinoff B.E., Pera M.F., Fong C.Y., Trounson A., Bongso A. (2000). Embryonic stem cell lines from human blastocysts: Somatic, differentiation *in vitro*. Nat. Biotechnol..

[43] Reubinoff B.E., Itsykson P., Turetsky T., Pera M.F., Reinhartz E., Itzik A., Ben-Hur T. (2001). Neural progenitors from human embryonic stem cells. Nat. Biotechnol..

[44] Schuldiner M., Eiges R., Eden A., Yanuka O., Itskovitz-Eldor J., Goldstein R.S., Benvenisty N. (2001). Induced neuronal differentiation of human embryonic stem cells. Brain Res..

[45] Schuldiner M., Yanuka O., Itskovitz-Eldor J., Melton D.A., Benvenisty N. (2000). Effects of eight growth factors on the differentiation of cells derived from human embryonic stem cells. Proc. Natl. Acad. Sci. USA.

[46] Zhang S.C., Wernig M., Duncan ID., Brustle O., Thomson J.A. (2001). *In vitro* differentiation of transplantable neural precursors from human embryonic stem cells. Nat. Biotechnol..

[47] Kaufman D.S., Hanson E.T., Lewis R.L., Auerbach R., Thomson J.A. (2001). Hematopoietic colony-forming cells derived from human embryonic stem cells. Proc. Natl. Acad. Sci. USA.

[48] Kehat I., Kenyagin-Karsenti D., Snir M., Segev H., Amit M., Gepstein A., Livne E., Binah O., Itskovitz-Eldor J., Gepstein L. (2001). Human embryonic stem cells can differentiate into myocytes with structural and functional properties of cardiomyocytes. J. Clin. Invest..

[49] Levenberg S., Golub J.S., Amit M., Itskovitz-Eldor J., Langer R. (2002). Endothelial cells derived from human embryonic stem cells. Proc. Natl. Acad. Sci. USA.

[50] Assady S., Maor G., Amit M., Itskovitz-Eldor J., Skorecki K.L., Tzukerman M. (2001). Insulin production by human embryonic stem cells. Diabetes.

[51] Fehrer C., Lepperdinger G. (2005). Mesenchymal stem cell aging. Exp. Gerontol..

[52] Muraglia A., Cancedda R., Quarto R. (2000). Clonal mesenchymal progenitors from human bone marrow differentiate in vitro according to a hierarchical model. J. Cell Sci..

[53] Koh C.J., Atala A. (2004). Tissue engineering, stem cells, and cloning: Opportunities for regenerative medicine. J. Am. Soc. Nephrol..

[54] Itskovitz-Eldor J., Schuldiner M., Karsenti D., Eden A., Yanuka O., Amit M., Soreq H., Benvenisty N. (2000). Differentiation of human embryonic stem cells into embryoid bodies compromising the three embryonic germ layers. Mol. Med..

[55] Zhang W.W., Zhang X.J., Liu H.X., Chen J., Ren Y.H., Huang D.G., Zou X.H., Xiao W. (2011). Cdk1 is required for the self-renewal of mouse embryonic stem cells. J. Cell Biochem..

[56] Neganova I., Vilella F., Atkinson S.P., Lloret M., Passos J.F., von Zglinicki T., O'Connor J.E., Burks D., Jones R., Armstrong L. (2011). An important role for CDK2 in G1 to S checkpoint activation and DNA damage response in human embryonic stem cells. Stem Cells.

[57] Kamiya D., Banno S., Sasai N., Ohgushi M., Inomata H., Watanabe K., Kawada M., Yakura R., Kiyonari H., Nakao K. (2011). Intrinsic transition of embryonic stem-cell differentiation into neural progenitors. Nature.

[58] Jiang Z., Adams GB., Hanash AM., Scadden DT., Levy RB. (2002). The contribution of cytotoxic and noncytotoxic function by donor T-cells that support engraftment after allogeneic bone marrow transplantation. Biol. Blood Marrow Transplant..

[59] Bossolasco P., Montemurro T., Cova L., Zangrossi S., Calzarossa C., Buiatiotis S., Soligo D., Bosari S., Silani V., Deliliers GL. (2006). Molecular and phenotypic characterization of human amniotic fluid cells and their differentiation potential. Cell Res..

[60] Ilancheran S., Michalska A., Peh G., Wallace E.M., Pera M., Manuelpillai U. (2007). Stem cells derived from human fetal membranes display multilineage differentiation potential. Biol. Reprod..

[61] Lakshmipathy U., Verfaillie C. (2005). Stem cell plasticity. Blood Rev..

[62] Cao F.J., Feng S.Q. (2009). Human umbilical cord mesenchymal stem cells and the treatment of spinal cord injury. Chin. Med. J. (Engl)..

[63] Tonlorenzi R., Della valle A., Schnapp E., Cossu G., Sampaolesi M. (2007). Isolation and characterization of mesoangioblasts from mouse, dog, and human tissues. Curr. Protoc. Stem Cell Biol..

[64] Martino S., D'Angelo F., Armentano I., Tiribuzi R., Pennacchi M., Dottori M., Mattioli S., Caraffa A., Cerulli G.G., Kenny J.M., Orlacchio A. (2009). Hydrogenated amorphous carbon nanopatterned film designs drive human bone marrow mesenchymal stem cell cytoskeleton architecture. Tissue Eng. Part A..

[65] Martino S., Tiribuzi R., Ciraci E., Makrypidi G., D'Angelo F., di Girolamo I., Gritti A., de Angelis G.M., Papaccio G., Sampaolesi M. (2011). Coordinated involvement of cathepsins S, D and cystatin C in the commitment of hematopoietic stem cells to dendritic cells. Int. J. Biochem. Cell Biol..

[66] Vescovi A.L., Snyder E.Y. (1999). Establishment and properties of neural stem cell clones: plasticity *in vitro and in vivo*. Brain Pathol..

[67] Lindvall O., Kokaia Z., Martinez-Serrano A. (2004). Stem cell therapy for human neurodegenerative disorders-how to make it work. Nat Med..

[68] Gritti A., Galli R., Vescovi A.L. (2008). Clonal analyses and cryopreservation of neural stem cell cultures. Methods Mol. Biol..

[69] Lee P.H., Park H.J. (2009). Bone marrow-derived mesenchymal stem cell therapy as a candidate disease-modifying strategy in Parkinson's disease and multiple system atrophy. J. Clin. Neurol..

[70] Orlacchio A., Bernardi G., Orlacchio A., Martino S. (2010). Stem cells: an overview of the current status of therapies for central and peripheral nervous system diseases. Curr. Med. Chem..

[71] Galli R., Borello U., Gritti A., Minasi M.G., Bjornson C., Coletta M., Mora M., De Angelis M.G., Fiocco R., Cossu G. (2000). Skeletal myogenic potential of human and mouse neural stem cells. Nat. Neurosci..

[72] Brittan M., Wright N.A. (2002). Gastrointestinal stem cells. J. Pathol..

[73] Lemischka I.R., Moore K.A. (2003). Stem cells: Interactive niches. Nature.

[74] Kiger A.A., Jones D.L., Schulz C., Rogers M.B., Fuller M.T. (2001). Stem cell self-renewal specified by JAK-STAT activation in response to a support cell cue. Science.

[75] Spradling A., Drummond-Barbosa D., Kai T. (2001). Stem cells find their niche. Nature.

[76] Cassano M., Quattrocelli M., Crippa S., Perini I., Ronzoni F., Sampaolesi M. (2009). Cellular mechanisms and local progenitor activation to regulate skeletal muscle mass. J. Muscle. Res. Cell Motil..

[77] d'Aquino R., Tirino V., Desiderio V., Studer M., De Angelis G.C., Laino L., De Rosa A., Di Nucci D., Martino S., Paino F. (2011). Human neural crest-derived postnatal cells exhibit remarkable embryonic attributes either *in vitro* or *in vivo*. Eur. Cell Mater..

[78] Parolini O., Caruso M. (2011). Review: Preclinical studies on placenta-derived cells and amniotic membrane: an update. Placenta..

[79] Jiang Y., Lv H., Huang S., Tan H., Zhang Y., Li H. (2011). Bone marrow mesenchymal stem cells can improve the motor function of a Huntington's disease rat model. Neurol Res..

[80] Marcus A.J., Woodbury D. (2008). Fetal stem cells from extra-embryonic tissues: Do not discard. J. Cell Mol. Med..

[81] Takahashi K., Yamanaka S. (2006). Induction of pluripotent stem cells from mouse embryonic and adult fibroblast cultures by defined factors. Cell.

[82] Hu K., Yu J., Suknuntha K., Tian S., Montgomery K., Choi K.D., Stewart R., Thomson J.A., Slukvin I.I. (2011). Efficient generation of transgene-free induced pluripotent stem cells from normal and neoplastic bone marrow and cord blood mononuclear cells. Blood.

[83] Montserrat N., Garreta Bahima E., Gonzalez F., Gutierrez J., Eguizabal C., Ramos Perez V., Borros S., Izpisua Belmonte J.C. (2011). Simple generation of human induced Pluripotent stem cells using Poly({beta}-Amino Esters) as non-viral gene delivery system. J. Biol. Chem..

[84] Narsinh K.H., Sun N., Sanchez-Freire V., Lee A.S., Almeida P., Hu S., Jan T., Wilson K.D., Leong D., Rosenberg J. (2011). Single cell transcriptional profiling reveals heterogeneity of human induced pluripotent stem cells. J. Clin. Invest..

[85] Pasi C.E., Dereli-Öz A., Negrini S., Friedli M., Fragola G., Lombardo A., van Houwe G., Naldini L., Casola S., Testa G., Halazonetis T.D. (2011). Genomic instability in induced stem cells. Cell Death Differ..

[86] Yazawa M., Hsueh B., Jia X., Pasca A.M., Bernstein J.A., Hallmayer J., Dolmetsch R.E. (2011). Using induced pluripotent stem cells to investigate cardiac phenotypes in Timothy syndrome. Nature.

[87] Ye Z., Zhan H., Mali P., Dowey S., Williams D.M., Jang Y.Y., Dang C.V., Spivak J.L., Moliterno A.R., Cheng L. (2009). Human-induced pluripotent stem cells from blood cells of healthy donors and patients with acquired blood disorders. Blood.

[88] Park I.H., Arora N., Huo H., Maherali N., Ahfeldt T., Shimamura A., Lensch M.W., Cowan C., Hochedlinger K., Daley G.Q. (2008). Disease-specific induced pluripotent stem cells. Cell.

[89] Ebert A.D., Yu J., Rose F.F., Mattis V.B., Lorson C.L., Thomson J.A., Svendsen C.N. (2009). Induced pluripotent stem cells from a spinal muscular atrophy patient. Nature.

[90] Vitale A.M., Wolvetang E., Mackay-Sim A. (2011). Induced pluripotent stem cells: A new technology to study human diseases. Int. J. Biochem. Cell Biol..

[91] Armentano I., Dottori M., Fortunati E., Mattioli S., Kenny J.M. (2010). Biodegradable polymer matrix nanocomposites for tissue engineering: A review. Polym. Degrad. Stabil..

[92] Wang J.H., Thampatty B.P. (2008). Mechanobiology of adult and stem cells. Int. Rev. Cell Mol. Biol..

[93] Wang N., Tytell J.D., Ingber D.E. (2009). Mechanotransduction at a distance: mechanically coupling the extracellular matrix with the nucleus. Nat. Rev. Mol. Cell Biol..

[94] del Rio A., Perez-Jimenez R., Liu R., Roca-Cusachs P., Fernandez J.M., Sheetz M.P. (2009). Stretching single talin rod molecules activates vinculin binding. Science.

[95] Ingber D.E. (2006). Cellular mechanotransduction: putting all the pieces together again. FASEB J..

[96] Ingber D.E. (2008). Tensegrity-based mechanosensing from macro to micro. Prog Biophys Mol Biol..

[97] Burridge K., Chrzanowska-Wodnicka M. (1996). Focal adhesions, contractility, and signalling. Annu. Rev. Cell Dev. Biol..

[98] Ingberg D. (1991). Integrins as mechanochemical transducers. Curr. Opin. Cell. Biol..

[99] Sadoshima J., Izumo S. (1997). the cellular and molecular response of myocytes to mechanical stress. Annu. Rev. Physiol..

[100] Jaalouk D.E., Lammerding J. (2009). Mechanotransduction gone away. Nat. Rev. Mol. Cell Biol..

[101] D'Angelo F., Armentano I., Mattioli S., Crispoltoni L., Tiribuzi R., Cerulli G.G., Palmerini C.A., Kenny J.M., Martino S., Orlacchio A. (2010). Micropatterned hydrogenated amorphous carbon guides mesenchymal stem cells towards neuronal differentiation. Eur. Cell Mater..

[102] Biggs M.J., Richards R.G., Dalby M.J. (2010). Nanotopographical modification: a regulator of cellular function through focal adhesions. Nanomedicine.

[103] Sardella E., Detomaso L., Gristina R., Senesi G.S., Agheli H., Sutherland D.S., d'Agostino R., Favia P. (2008). Nano-structured cell-adhesive and cell-repulsive plasma-deposited coatings: chemical and topographical effects on keratinocyte adhesion. Plasma Process. Polym..

[104] Armentano I., Ciapetti G., Pennacchi M., Dottori M., Devescovi V., Granchi D., Baldini N., Olalde B., Jurado M.J., Marquinez Alava J.I. (2009). Role of PLLA Plasma Surface Modification in the Interaction with Human Marrow Stromal Cells. J. Appl. Polym. Sci..

[105] Di Mundo R., Gristina R., Sardella E., Intranuovo F., Nardulli M., Milella A., Palumbo F., d'Agostino R., Favia P. (2010). Micro-/nanoscale structuring of cell-culture substrates with fluorocarbon plasmas. Plasma Process. Polym..

[106] Plant A.L., Bhadriraju K., Spurlin T.A., Elliott J.T. (2009). Review Cell response to matrix mechanics: Focus on collagen. Biochim. Biophys. Acta..

[107] Pek Y.S., Wan A.C.A., Ying J.Y. (2010). The effect of matrix stiffness on mesenchymal stem cell differentiation in a 3D thixotropic gel. Biomaterials.

[108] Yeung T., Georges P.C., Flanagan L.A., Marg B., Ortiz M., Funaki M., Zahir N., Ming W., Weaver V., Janmey P.A. (2005). Effects of substrate stiffness on cell morphology, cytoskeletal structure, and adhesion. Cell Motil. Cytoskeleton..

[109] Akhyari P., Fedak P.W., Weisel R.D., Lee T.Y., Verma S., Mickle D.A., Li R.K. (2002). Mechanical stretch regimen enhances the formation of bioengineered autologous cardiac muscle grafts. Circulation.

[110] Garvin J., Qi J., Maloney M., Banes A.J. (2003). Novel system for engineering bioartificial tendons and application of mechanical load. Tissue Eng..

[111] Dalby M.J., Gadegaard N., Tare R., Andar A., Riehle M.O., Herzyk P., Wilkinson C.D., Oreffo R.O. (2007). The control of human mesenchymal cell differentiaiton using nanoscale symmetry and disorder. Nat. Mater..

[112] Engler A.J., Sen S., Sweeney H.L., Discher D.E. (2006). Matrix elasticity directs stem cell lineage specification. Cell.

[113] Leipzig N.D., Shoichet M.S. (2009). The effect of substrate stiffness on adult neural stem cell behavior. Biomaterials.

[114] Huebsch N., Arany P.R., Mao A.S., Shvartsman D., Ali O.A., Bencherif S.A., Rivera-Feliciano J., Mooney D.J. (2010). Harnessing traction-mediated manipulation of the cell/matrix interface to control stem-cell fate. Nat. Mater..

[115] Kilian K.A., Bugarija B., Lahn B.T., Mrksich M. (2010). Geometric cues for directing the differentiation of mesenchymal stem cells. Proc. Natl. Acad. Sci. USA..

[116] Ingber D. (2002). Mechanical signaling. Ann. N Y Acad. Sci..

[117] Katsumi A., Orr A.W., Tzima E., Schwartz M.A. (2004). Integrins in mechanotransduction. J. Biol. Chem..

[118] Schwartz M.A., Schaller M.D., Ginsberg M.H. (1995). Integrins: Emerging paradigms of signal transduction. Annu. Rev. Cell Dev. Biol..

[119] Wang N., Butler J.P., Ingber D.E. (1993). Mechanotransduction across the cell surface and through the cytoskeleton. Science.

[120] Ingber D.E. (1993). Cellular tensegrity: Defining new rules of biological design that govern the cytoskeleton. J Cell Sci..

[121] Stupack D.G. (2007). The biology of integrins. Oncology (Williston Park).

[122] Geiger B., Bershadsky A. (2002). Exploring the neighborhood: adhesion-coupled cell mechanosensors. Cell.

[123] Sawada Y., Sheetz M.P. (2002). Force transduction by triton cytoskeletons. J. Cell Biol..

[124] Wang N., Naruse K., Stamenović D., Fredberg J.J., Mijailovich S.M., Tolić-Nørrelykke I.M., Polte T., Mannix R., Ingber D.E. (2001). Mechanical behavior in living cells consistent with the tensegrity model. Proc. Natl. Acad. Sci. USA..

[125] Kamm K.E., Stull J.T. (2001). Dedicated myosin light chain kinases with diverse cellular functions. J. Biol. Chem..

[126] Pfitzer G. (2001). Invited review: Regulation of myosin phosphorylation in smooth muscle. J. Appl. Physiol..

[127] Chrzanowska-Wodnicka M., Burridge K. (1996). Rho-stimulated contractility drives the formation of stress fibers and focal adhesions. J. Cell Biol..

[128] Bershadsky A.D., Ballestrem C., Carramusa L., Zilberman Y., Gilquin B., Khochbin S., Alexandrova A.Y., Verkhovsky A.B., Shemesh T., Kozlov M.M. (2006). Assembly and mechanosensory function of focal adhesions: experiments and models. Eur. J. Cell Biol..

[129] Geiger P.C., Bailey J.P., Mantilla C.B., Zhan W.Z., Sieck G.C. (2006). Mechanisms underlying myosin heavy chain expression during development of the rat diaphragm muscle. J. Appl. Physiol..

[130] Ingber D.E. (2003). Mechanobiology and diseases of mechanotransduction. Ann. Med..

[131] Kumar S., Maxwell I.Z., Heisterkamp A., Polte T.R., Lele T.P., Salanga M., Mazur E., Ingber D.E. (2006). Viscoelastic retraction of single living stress fibers and its impact on cell shape, cytoskeletal organization, and extracellular matrix mechanics. Biophys. J..

[132] Brangwynne C.P., MacKintosh F.C., Kumar S., Geisse N.A., Talbot J., Mahadevan L., Parker K.K., Ingber D.E., Weitz D.A. (2006). Microtubules can bear enhanced compressive loads in living cells because of lateral reinforcement. J. Cell Biol..

[133] Vikstrom K.L., Lim S.S., Goldman R.D., Borisy G.G. (1992). Steady state dynamics of intermediate filament networks. J. Cell Biol..

[134] Fey E.G., Wan K.M., Penman S. (1984). Epithelial cytoskeletal framework and nuclear matrix-intermediate filament scaffold: Three-dimensional organization and protein composition. J. Cell Biol..

[135] Maniotis A.J., Chen C.S., Ingber D.E. (1997). Demonstration of mechanical connections between integrins, cytoskeletal filaments, and nucleoplasm that stabilize nuclear structure. Proc. Natl. Acad. Sci. USA.

[136] Wang N., Tytell J.D., Ingber D.E. (2009). Mechanotransduction at a distance: Mechanically coupling the extracellular matrix with the nucleus. Nat. Rev. Mol. Cell Biol..

[137] Kang Y.G., Nam J.H., Kim K.H., Lee K.S. (2010). FAK pathway regulates PGE production in compressed periodontal ligament cells. J. Dent Res..

[138] Ziegler N., Alonso A., Steinberg T., Woodnutt D., Kohl A., Müssig E., Schulz S., Tomakidi P. (2010). Mechano-transduction in periodontal ligament cells identifies activated states of MAP-kinases p42/44 and p38-stress kinase as a mechanism for MMP-13 expression. BMC Cell Biol..

[139] Provenzano P.P., Inman D.R., Eliceiri K.W., Keely P.J. (2009). Matrix density-induced mechanoregulation of breast cell phenotype, signaling and gene expression through a FAK-ERK linkage. Oncogene.

[140] Wu Z., Wong K., Glogauer M., Ellen R.P., McCulloch C.A. (1999). Regulation of stretch-activated intracellular calcium transients by actin filaments. Biochem. Biophys. Res. Commun..

[141] Iqbal J., Zaidi M. (2005). Molecular regulation of mechanotransduction. Biochem. Biophys. Res. Commun..

[142] Rosen L.B., Greenberg M.E. (1996). Stimulation of growth factor receptor signal transduction by activation of voltage-sensitive calcium channels. Proc. Natl. Acad. Sci. USA..

[143] Sadoshima J., Izumo S. (1997). The cellular and molecular response of cardiac myocytes to mechanical stress. Annu. Rev. Physiol..

[144] Sarasa-Renedo A., Chiquet M. (2005). Mechanical signals regulating extracellular matrix gene expression in fibroblasts. Scand. J. Med. Sci. Sports..

[145] White C.R., Frangos J.A. (2007). The shear stress of it all: the cell membrane and mechanochemical transduction. Philos. Trans. R Soc. Lond. B Biol. Sci..

[146] Vogel V., Sheetz M. (2006). Local force and geometry sensing regulate cell functions. Nat. Rev. Mol. Cell Biol..

[147] Chien S. (2007). Mechanotransduction and endothelial cell homeostasis: the wisdom of the cell. Am. J. Physiol. Heart Circ. Physiol..

[148] Hahn C., Schwartz M.A. (2009). Mechanotransduction in vascular physiology and atherogenesis. Nat. Rev. Mol. Cell Biol..

[149] Tzima E., Irani-Tehrani M., Kiosses W.B., Dejana E., Schultz D.A., Engelhardt B., Cao G., DeLisser H., Schwartz M.A. (2005). A mechanosensory complex that mediates the endothelial cell response to fluid shear stress. Nature.

[150] Shi Z.D., Wang H., Tarbell J.M. (2011). Heparan sulfate proteoglycans mediate interstitial flow mechanotransduction regulating MMP-13 expression and cell motility via FAK-ERK in 3D collagen. PLoS One..

[151] Sinha B., Köster D., Ruez R., Gonnord P., Bastiani M., Abankwa D., Stan R.V., Butler-Browne G., Vedie B., Johannes L., Morone N., Parton R.G., Raposo G., Sens P., Lamaze C., Nassoy P. (2011). Cells respond to mechanical stress by rapid disassembly of caveolae. Cell.

[152] Wang Y., Maciejewski B.S., Drouillard D., Santos M., Hokenson M.A., Hawwa R.L., Huang Z., Sanchez-Esteban J. (2010). A role for caveolin-1 in mechanotransduction of fetal type II epithelial cells. Am. J. Physiol. Lung Cell Mol. Physiol..

[153] Yang Z., Bidwell J.P., Young S.R., Gerard-O'Riley R., Wang H., Pavalko F.M. (2010). Nmp4/CIZ inhibits mechanically induced beta-catenin signaling activity in osteoblasts. J. Cell Physiol..

[154] Jansen J.H., Eijken M., Jahr H., Chiba H., Verhaar J.A., van Leeuwen J.P., Weinans H. (2010). Stretch-induced inhibition of Wnt/beta-catenin signaling in mineralizing osteoblasts. J. Orthop. Res..

[155] Santos A., Bakker A.D., Zandieh-Doulabi B., de Blieck-Hogervorst J.M., Klein-Nulend J. (2010). Early activation of the beta-catenin pathway in osteocytes is mediated by nitric oxide, phosphatidyl inositol-3 kinase/Akt, and focal adhesion kinase. Biochem. Biophys. Res. Commun..

[156] Liedert A., Wagner L., Seefried L., Ebert R., Jakob F., Ignatius A. (2010). Estrogen receptor and Wnt signaling interact to regulate early gene expression in response to mechanical strain in osteoblastic cells. Biochem. Biophys. Res. Commun..

[157] Subbaiah R., Thomas B. (2011). Efficacy of a bioactive alloplast, in the treatment of human periodontal osseous defects-a clinical study. Med. Oral Patol. Oral Cir. Bucal..

[158] Sendtner M. (2009). Stem cells: Tailor-made diseased neurons. Nature.

[159] Soldner F., Hockemeyer D., Beard C., Gao Q., Bell G.W., Cook E.G., Hargus G., Blak A., Cooper O., Mitalipova M. (2009). Parkinson's disease patient-derived induced pluripotent stem cells free of viral reprogramming factors. Cell.

[160] Dimos J.T., Rodolfa K.T., Niakan K.K., Weisenthal L.M., Mitsumoto H., Chung W., Croft G.F., Saphier G., Leibel R., Goland R. (2008). Induced pluripotent stem cells generated from patients with ALS can be differentiated into motor neurons. Science.

